# Spatial and temporal structure of diversity and demographic dynamics along a successional gradient of tropical forests in southern Brazil

**DOI:** 10.1002/ece3.5816

**Published:** 2020-03-13

**Authors:** Cilmar Antônio Dalmaso, Marcia C. M. Marques, Pedro Higuchi, Victor P. Zwiener, Renato Marques

**Affiliations:** ^1^ Programa de Pós‐graduação em Engenharia Florestal Universidade Federal do Paraná Curitiba Brazil; ^2^ Departamento de Botânica SCB Laboratório de Ecologia Vegetal Universidade Federal do Paraná Curitiba Brazil; ^3^ Departamento de Engenharia Florestal Universidade do Estado de Santa Catarina, Centro de Ciências Agroveterinárias Lages Brazil; ^4^ Departamento de Biodiversidade Universidade Federal do Paraná Palotina Brazil; ^5^ Departamento de Solos e Engenharia Agrícola Laboratório de Biogeoquímica Universidade Federal do Paraná Curitiba Brazil

**Keywords:** beta diversity, biodiversity conservation, floristic richness, nestedness, structural change, Submontane Dense Ombrophilous Forest, turnover

## Abstract

1. Analysis of the structure, diversity, and demographic dynamics of tree assemblages in tropical forests is especially important in order to evaluate local and regional successional trajectories.
2. We conducted a long‐term study to investigate how the structure, species richness, and diversity of secondary tropical forests change over time. Trees (DBH ≥ 5 cm) in the Atlantic Forest of southern Brazil were sampled twice during a 10‐year period (2007 and 2017) in six stands (1 ha each) that varied in age from their last disturbance (25, 60, 75, 90, and more than 100 years). We compared forest structure (abundance and basal area), species richness, alpha diversity, demographic rates (mortality, recruitment, and loss or gain in basal area), species composition, spatial beta diversity, and temporal beta diversity (based on turnover and nestedness indices) among stand ages and study years.
3. Demographic rates recorded in a 10‐year interval indicate a rapid and dynamic process of species substitution and structural changes. Structural recovery occurred faster than beta diversity and species composition recovery. The successional gradient showed a pattern of species trade‐off over time, with less spatial dissimilarity and faster demographic rates in younger stands. As stands grow older, they show larger spatial turnover of species than younger stands, making them more stochastic in relation to species composition. Stands appear to split chronologically to some extent, but not across a straightforward linear axis, reflecting stochastic changes, providing evidence for the formation of a nonequilibrium community.
4. *Policy implications*. These results reiterate the complexity and variability in forest succession and serve as a reference for the evaluation and monitoring of local management and conservation actions and for defining regional strategies that consider the diversity of local successional trajectories to evaluate the effectiveness of restoration measures in secondary forests of the Atlantic Forest biome.

## Graphical abstract

A long‐term study of changes in the structure and diversity of secondary forests. Effects of partitioning beta diversity of tropical secondary forests in Brazil. Reference work for the monitoring of recovery actions in tropical ecosystems.

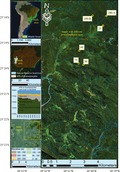

## 1 INTRODUCTION

With the widespread loss of primary forests in the tropics (Keenan et al., 2015), secondary forests now occupy a growing proportion of forest cover globally and are considered as valuable for maintaining biodiversity (Brook, Bradshaw, Koh, & Sodhi, 2006; Chazdon et al., 2009; Gardner, Barlow, Parry, & Peres, 2007; Laurance, 2007), providing ecosystem services, and supplying forest products (Chazdon & Coe, 1999; Ferraz et al., 2014; Foley et al., 2007). Considering that forest resilience is associated with history, evolutionary pressures, and previous extinctions, and that these factors only increase in scale, conservation actions should target entire communities rather than species (Balmford, 1996). In this context, long‐term studies are especially important in order to evaluate the effects of multiple factors affecting species assemblage over time and to provide more accurate future estimates of biodiversity conservation and ecosystem services by targeting current management and conservation actions (Gross et al., 2018; Liebsch, Marques, & Goldenberg, 2008; Marques, Burslem, Britez, & Silva, 2009; Phillips & Gentry, 1994; Phillips, Hall, Gentry, Sawyer, & Vásquez, 1994; Phillips et al., 1998; Sheil, 1999; Sheil, Jennings, & Savill, 2000).

After a disturbance, the structure and composition of a forest community changes over time, going through defined stages of the successional process (Arroyo‐Rodríguez et al., 2017; Chanthorn, Hartig, & Brockelman, 2017; Guariguata & Ostertag, 2001). The successional trajectory can be influenced by several factors, such as type and intensity of disturbance, and distance from the sources of propagules (Chazdon, 2008; Glenn‐Lewin, Peet, & Veblen, 1992; Hooper, Legendre, & Condit, 2004; Kauano, Cardoso, Torezan, & Marques, 2013). Some forest parameters, such as accumulated aboveground biomass and species richness, may recover rapidly within decades (25%–85% biomass and 80% species richness after two decades), while others, such as species composition, may require centuries (Poorter et al., 2016; Rozendaal et al., 2019). Species can lead to variations in demographic rates especially when species respond differently to environmental changes in autoecology, phylogenetics, physiological activity, leaf longevity, and allometric relationships between biomass of leaves stems and roots (Givnish, 2002; Poorter et al., 2012). Additionally, the dynamics of vegetation during succession of the secondary rainforest reflects a complex interaction between deterministic and stochastic processes (Chazdon, 2008). It is a multifactorial phenomenon with different possible trajectories (Arroyo‐Rodríguez et al., 2017; Chazdon et al., 2009). That is, dynamic and diversity patterns depend on the relative strength of deterministic processes versus those of stochastic processes. For example, it is possible to have predictive rates of species renewal throughout the successional process (Cequinel, Capellesso, Marcilio‐Silva, Cardoso, & Marques, 2018), although species compositions may vary stochastically among forest communities (Norden et al., 2015). For temporal patterns to be synchronized within a habitat, there must be an environmental filter that consistently determines the relative strength of stochastic versus deterministic dynamics (Van Allen, Rasmussen, Dibble, Clay, & Rudolf, 2017). Assessing such trajectories is an important step for establishing efficient local management and conservation strategies, and for defining regional strategies that consider the diversity of local successional trajectories, including the monitoring of invasions and extinctions and taxonomic and functional simplification (Mckinney & Lockwood, 1999; Olden, Lockwood, & Parr, 2011; Solar et al., 2015).

Natural communities are not constant and can change substantially seasonally or on short‐ and long‐term scales (Magurran & Dornelas, 2010; Van Allen et al., 2017). In successional studies of tropical landscapes, it still remains unclear how population and species turnovers change over space and time and with the spatial and/or temporal scale (Condit et al., 2002). Even considering that mortality and recruitment rates are dynamic in tropical forests (Gomes, Mantovani, & Kageyama, 2003; Korning & Balslev, 1994; Marques et al., 2009; Phillips & Gentry, 1994) and that migration processes are slower and dependent on landscape attributes (Arroyo‐Rodríguez et al., 2017; Chazdon, 2008), species replacement rates are expected to drive diversity. In this context, the partitioning of the beta diversity can be particularly useful and might help to identify potential biological processes that may be relevant in structuring the diversity of tropical forest communities and how these processes change over time (Baselga, 2010). The total spatial dissimilarity is partitioned into two components (turnover and nestedness) based on the Sorensen (βsor) and Simpson (βsim) indices. βsim represents the spatial turnover of species, while βsne (obtained by the difference between βsor and βsim) shows the loss or gain of species due to nestedness (Baselga, 2010). When both localities have the same number of species, β_sor_ and β_sim_ are the same and any dissimilarity between two localities with the same number of species is completely due to spatial rotation (*turnover*), because nestedness can not occur. The total temporal beta diversity is divided in the same way, considering two periods for the same locality. Partitioning of beta diversity into nestedness and turnover can help us understand the selective differentiation among sites, which is useful for analyzing the causality of the processes underlying biodiversity changes (Baselga, 2010; Wright & Reeves, 1992). Nestedness may lead to the disintegration of assemblages by the replication of subsets of species, as opposed to turnover, which is a natural process in which species are replaced by environmental selection, or historical and spatial constraints (Baselga & Orme, 2012).

In the Atlantic Forest of Brazil, differences in historical disturbances resulted in fragments of secondary forests that are in different stages of recovery but have a large proportion of their biodiversity conserved (Cruz et al., 2018; Ribeiro, Metzger, Martensen, Ponzoni, & Hirota, 2009; Salami et al., 2017). Under this scenario, to improve conservation and management actions for these forest remnants, we conducted a long‐term study in secondary forests in the southern region of the Atlantic Forest to investigate how these forests changed in time and space in relation to structure (abundance and basal area), floristic richness, species composition, demographic dynamics rates, alpha diversity and spatial beta diversity. Our study was carried out over a 10‐year period (2007–2017) in the stands of secondary forests that were disturbed by total and/or selective logging at different times in the past (25, 60, 75, 90, and 100 years ago) to specifically test the following hypotheses: (1) The stands differ more predictably in floristic richness, diversity, structural parameters (abundance and basal area), while rates of population dynamics, species composition, and beta diversity (temporal and spatial) are more stochastic; (2) Spatial beta diversity and its turnover component increase with stand age, while nestedness decreases and an inverse effect is expected with little predictability for temporal beta diversity and its respective components; (3) The younger stands (25, 60, and 75) present higher and more stochastic demographic rates than the older stands (90, 100A, and 100B).

## 2 MATERIALS AND METHODS

### 2.1 Study area and sample design

The study was conducted on the slopes of the Serra do Mar, in the municipality of Antonina, Paraná State, Brazil (Figure S1). The region is inside a large continuous forest‐covered massif that extends across a mountainous and well‐preserved landscape from Parana State to São Paulo State. The region has a set of conservation units where the environmental protection areas of Guaraqueçaba, which cover several conservation units, are inserted. The study site is located in the Guaricica Natural Reserve (48°40′W and 25°19′S), a private protected area that has been under environmental protection for 18 years and covers 8,600 ha. The reserve comprises areas with elevations varying from 3 to 500 m within the Lowland Ombrophilous Forest, Submontane Ombrophilous Forest, and Montane Ombrophilous Forest.

Six, 1 ha stands (plots of 100 × 100 m, subdivided into 20 × 20 m subplots) were established within secondary forests at elevation ranging from 10 to 180 m above sea level (Submontane Ombrophilous Forest). All stands were located within a radius of approximately 2 km, with the minimum distance between stands being 250 m. The age of each stand in terms of the time elapsed since the last disturbance was estimated from reports by residents, aerial imaging, and dendrochronological studies (Gobel, 2016). The youngest stand, estimated to be 25 years of age (Stand‐25), was previously cultivated for agriculture and had soil exposed for 30 years. The initial stage of succession reached only 17 years before this study (2000). The stand estimated to be 60 years of age (Stand‐60) had dense forests that started an early stage of succession 37 years ago (1980). The stand estimated to be 75 years of age (Stand‐75) began the initial stage of forest succession 65 years ago (1952). The stand estimated to be 90 years of age (Stand‐90) had part of its coverage at the initial stage of forest succession 65 years ago (1952), and the remainder was covered with a dense forest. Two areas with secondary vegetation estimated to be more than 100 years of age (Stand‐100A and Stand‐100B), had a dense forest as shown in photographs taken since 1952 (65 years ago), but there was selective logging in these areas (according to reports from former local workers) close to old roads that were used to transport logged wood from the forest.

The soils of all areas include Dystric Acrisol and Cambisol in Stand‐25, Stagnic Dystric Gleysol and Dystric Cambisol in Stand‐60, Dystric Cambisol in Stand‐75, Stand‐90, and Stand‐100A; and Dystric Acrisol and Dystric Cambisol in Stand‐100B (Gobel, 2016; IUSS Working Group WRB, 2015). All soils are characterized by high acidity, high concentrations of C, and low or very low levels of nutrients (P, K, Ca, and Mg) in the 0–20‐cm layer (Woiciechowski & Marques, 2017).

### 2.2 Data collection

The first data collection was in 2007, and all arboreal and arborescent trees with a diameter at breast height (DBH) ≥5 cm were identified, numbered with metallic tags, and their DBH measured. During the second sampling (2017), all trees were reevaluated to record mortality, measure and identify recruits, and remeasure survivors in 150 subplots of 0.04 ha each, which comprised six hectares of secondary forest.

### 2.3 Demographic rates

For each subplot, we calculated the annual mortality and recruitment rates, by considering, respectively, the number of living trees tagged in 2007 and those not found or dead in 2017, and the number of recruits (i.e., trees without numbered tags). The turnover rate, assessed in terms of trees abundance, represents the annual average rate of mortality and recruitment. Additionally, we calculated the basal area loss rate considering basal area of dead trees and the eventual decrease in survivors, and basal area gain rate considering basal area gained from recruits and from the increase in the basal area of survivors. We calculated the basal area turnover rate as the mean annual difference between the basal area loss rate and the basal area gain rate. The demographic rates were calculated following Phillips and Gentry (1994), Oliveira‐Filho et al. (2007), Sheil, Burslem, and Alder (1995), and Sheil et al. (2000) (Table S1). All demographic rates were estimated using equations programmed into the R statistical programing language by Higuchi (2017). In practice, it can be assumed that within the period 2007–2017, demography was stationary, because annual surveys between 2007 and 2017 were not possible.

### 2.4 Analysis

Potential effects of stands on the species richness, alpha diversity, abundance, basal area, demographic rates (mortality, recruitment, turnover of abundance, loss rate in basal area, gain rate in basal area, and turnover of basal area), beta spatial diversity (turnover, nestedness, and turnover + nestedness), and temporal changes in community composition over ten years according to the components of beta diversity (turnover, nestedness, and turnover + nestedness) were tested with a two‐way ANOVA with permutation, using statistical functions of the “lmPerm” package in R (R Core Team, 2019; Wheeler & Torchiano, 2016). The time was defined categorically (two levels, 2007 and 2017) as a covariate to control for temporal heterogeneity. The results that indicated significant variations in stands were subjected to Fisher's test using Bonferroni correction with *p* < .05, using functions from the R package “agricolae” (Mendiburu, 2017; R Core Team, 2019).

The patterns of species composition were compared among the different stands and time (2007 and 2017) through a nonmetric multidimensional scaling (nMDS) ordination. The nMDS was performed using a distance matrix of Bray–Curtis dissimilarity values with the function “metaMDS,” with 2, 3, and 4 dimensions and 100 iterations, while the multivariate homogeneity of group dispersion was tested with the function “betadisper” and PERMANOVA was applied using 999 permutations with the statistical function “adonis,” both in the “vegan” package in R (Oksanen et al., 2019; R Core Team, 2019); comparison between groups was done with Tukey's “Honest Significant Difference” method.

We analyzed the changes in species composition using the beta diversity (β) approach proposed by Baselga (2012), where total dissimilarity is partitioned into two components (turnover and nestedness) based on the Sørensen (β_sor_) (Sorensen, 1948) and Simpson (β_sim_) (Koleff, Gaston, & Lennon, 2003) indices (Table S1). β_sim_ represents the spatial turnover of species, while β_sne_ (obtained from the difference between β_sor_ and β_sim_) represents the loss or gain of species due to nestedness (Baselga, 2010). We calculated the turnover and nestedness at the stand level (differences among plots) and time level (differences between 2007 and 2017). The analyses were performed in the statistical programing language R (R Core Team, 2019), using the “beta‐multi.R,” “beta‐sample.R,” “beta‐temp.R” functions of the “betapart” package (Baselga, Orme, Villeger, Bortoli, & Leprieur, 2018). While the R function “beta‐multi” computes multiple‐site dissimilarities accounting for the spatial turnover and the nestedness components of beta diversity, plus the sum of both values, the “beta‐temp” function does the same for each locality between study years (time 1 and time 2). We used the function “beta‐sample” to resample multiple‐site dissimilarities controlling the number of sites in 10 with 100 replicates, and we generated the distribution of values into “beta‐multi” (Baselga & Orme, 2012). Without generating the distribution of values with “beta‐sample” there would only be a single value for each component of beta diversity, and it would not be possible to describe and compare the variability and dispersion of the data. For beta‐temp, the distribution of the values was generated using the results for the pairwise (2007–2017) calculation of the 25 subplots in each stand.

## 3 RESULTS

### 3.1 General stand structure and species richness

A total of 10,628 living arboreal trees of 212 species were sampled in the first survey (2007), and 10,170 trees of 215 species were sampled in the second survey (2017) (Table 1). Large differences among stands in terms of species richness (70–137 species), abundance (1,485–2,132 individuals), and basal area (25.56–36.49 m^2^/ha) were also observed (Table 1).

**Table 1:** Demography of secondary forests in southern Brazil (AB = abundance; BA = basal area)

Stand structural parameters and demographic rates	Stand postdisturbance age (years)						
	25	60	75	90	100A	100B	Total (6 ha)
Richness 2007 (species/ha)	70	100	130	133	125	137	212
Richness 2017 (species/ha)	89	95	128	130	132	132	215
Shannon 2007	2.61	3.51	3.63	3.46	3.85	4.11	4.20
Shannon 2017	2.74	3.53	3.59	3.44	3.86	4.03	4.16
Abundance 2007 (ind/ha)	2,132	1,722	1,927	1,604	1,624	1,619	10,628
Abundance 2017 (ind/ha)	2,115	1,573	1,867	1,601	1,529	1,485	10,170
BA 2007 (m^2^/ha)	22.7	31.02	33.28	32.23	32.35	32.87	184.45
BA 2017 (m^2^/ha)	25.56	32.93	36.49	35.91	36.06	34.88	201.83
Dead (ind/ha)	494	283	274	249	228	235	1,763
Recruits (ind/ha)	477	134	214	246	133	101	1,305
Mortality rate (% per year)	2.7	1.8	1.5	1.7	1.5	1.6	1.8
Recruitment rate (% per year)	2.6	1.0	1.2	1.7	1.0	0.7	1.36
AB Turnover (% per year)	2.7	1.4	1.4	1.7	1.2	1.2	1.58
BA Gain, survivors (m^2^/ha)	6.94	6.31	7.24	7.89	7.37	5.45	41.12
BA Dead (m^2^/ha)	5.44	4.56	4.52	5.08	3.95	3.61	27.16
BA Recruits (m^2^/ha)	1.71	0.47	0.66	1.11	0.45	0.28	4.68
BA Loss rate (% per year)	3.0	1.7	1.6	1.9	1.4	1.3	2.37
BA Gain rate (% per year)	4.2	2.3	2.5	3	2.4	1.8	2.55
BA Turnover (% per year)	3.6	2.0	2.0	2.4	1.9	1.5	2.46

**Figure 1:**
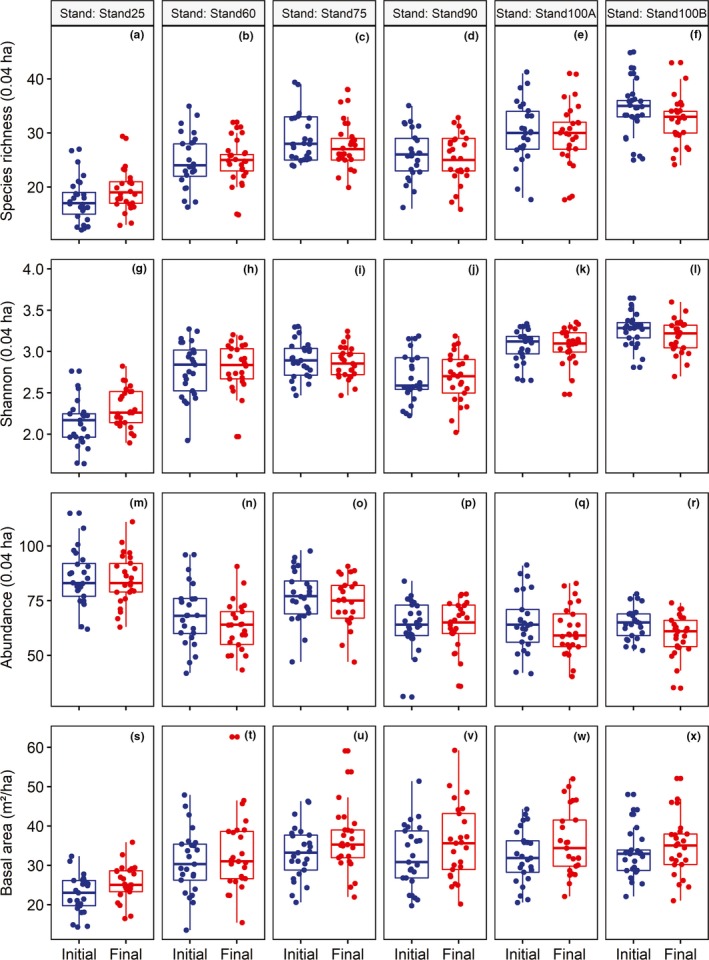
Species richness (a to f), Shannon alpha diversity index (g to l), abundance (m to r), and basal area (s to x) in 2007 and 2017 of six stand ages (25, 60, 75, 90, 100A, and 100B) of secondary forests in southern Brazil. Boxplots represent the medians and quartiles with the lower and upper limits

Species richness (Figure 1a–f) and alpha diversity (Figure 1g–l) were affected by stand age (Table 2), with a gradual increase in the number of species from Stand‐25 to Stand‐100A and Stand‐100B. Abundance was affected by stand age; there was a decrease in abundance from Stand‐25 to Stand‐100 (A and B) and from 2007 to 2017 (Figure 1m–r). Finally, there was an effect of stand age and study year on the basal area, which increased from Stand‐25 to Stand‐100 (A and B) and from 2007 to 2017 (Figure 1s–x).

**Table 2:** Two‐way ANOVA with permutations for the effects of stand age and study year on species richness and structural parameters (abundance, basal area)

	*df*	Sum Sq	Mean Sq	*p*
Species richness				
Stand age	5	6,693.4	1,338.67	<.001***
Residuals	294	6,095.8	20.7	
Study year	1	24.1	24.08	.234
Residuals	298	12,765.0	42.84	
Shannon alpha diversity				
Stand age	5	30.07	6.014	<.001***
Residuals	294	18.18	0.062	
Study year	1	0.014	0.014	.8824
Residuals	298	48.234	0.1619	
Abundance				
Stand age	5	20,878	4,175.7	<.001***
Residuals	294	38,348	130.4	
Study year	1	699	699.2	.034*
Residuals	298	58,527	196.4	
Basal area				
Stand age	5	4,133.3	826.67	<.001***
Residuals	294	17,175.3	58.42	
Study year	1	631.4	631.35	<.001***
Residuals	298	20,677.3	69.39	
Turnover				
Stand age	5	2.1772	0.4354	<.001***
Residuals	1,194	0.4175	0.0003	
Study year	1	0.0046	0.0046	.246
Residuals	1,198	2.5901	0.0022	
Nestedness				
Stand age	5	0.2790	0.0556	<.001***
Residuals	1,194	0.1628	0.0001	
Study year	1	0.0045	0.0045	<.001***
Residuals	1,198	0.4373	0.0003	
Nestedness + Turnover				
Stand age	5	0.95994	0.1920	<.001***
Residuals	1,194	0.17708	0.0001	
Study year	1	0.000	1.90e−07	1
Residuals	1,198	1.137	9.49e−04	

Significant codes: * is *p* < .05; *** is *p* < .001.

### 3.2 Demographic rates

**Figure 2:**
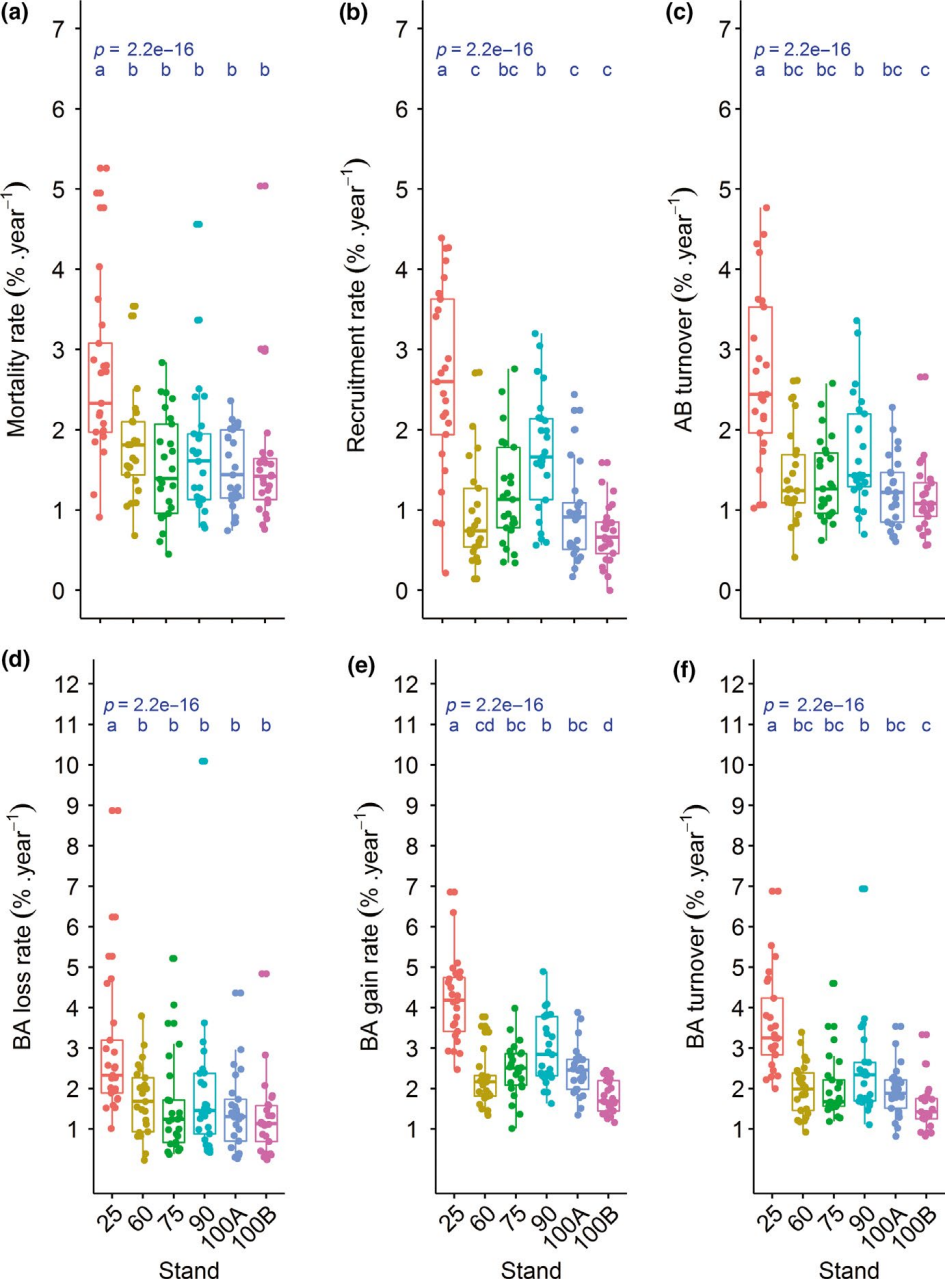
Demography rates of six stand ages (25, 60, 75, 90, 100A, and 100B) of secondary forests in southern Brazil. (a) mortality rate; (b) recruitment rate; (c) turnover of abundance; (d) loss rate in basal area; (e) gain rate in basal area; (f) turnover of basal area. AB, Abundance; BA, Basal area

A total of 1,763 trees died, and 1,305 trees were recruited during the 10‐year interval, with large variations in death and recruitment being observed among stands (228–494 and 101–477, respectively) during this time (Table 1). In general, the mortality rate was 1.8% per year, and the recruitment rate was 1.4% per year, indicating a turnover of approximately 1.6% per year (Table 1). There were differences among stands in terms of mortality, recruitment, and turnover rates (Figure 2a–c). In all cases, the younger stand (Stand‐25) presented a higher demographic rate (Figure 2a–c); however, no or small differences were observed among the older stands (Stand‐60 to Stand‐100B). In addition, the rates of basal area changes (loss, gain, and turnover) were higher in Stand‐25; with no or small differences being observed among other the stands (Figure 2d–f).

### 3.3 Species composition

**Figure 3:**
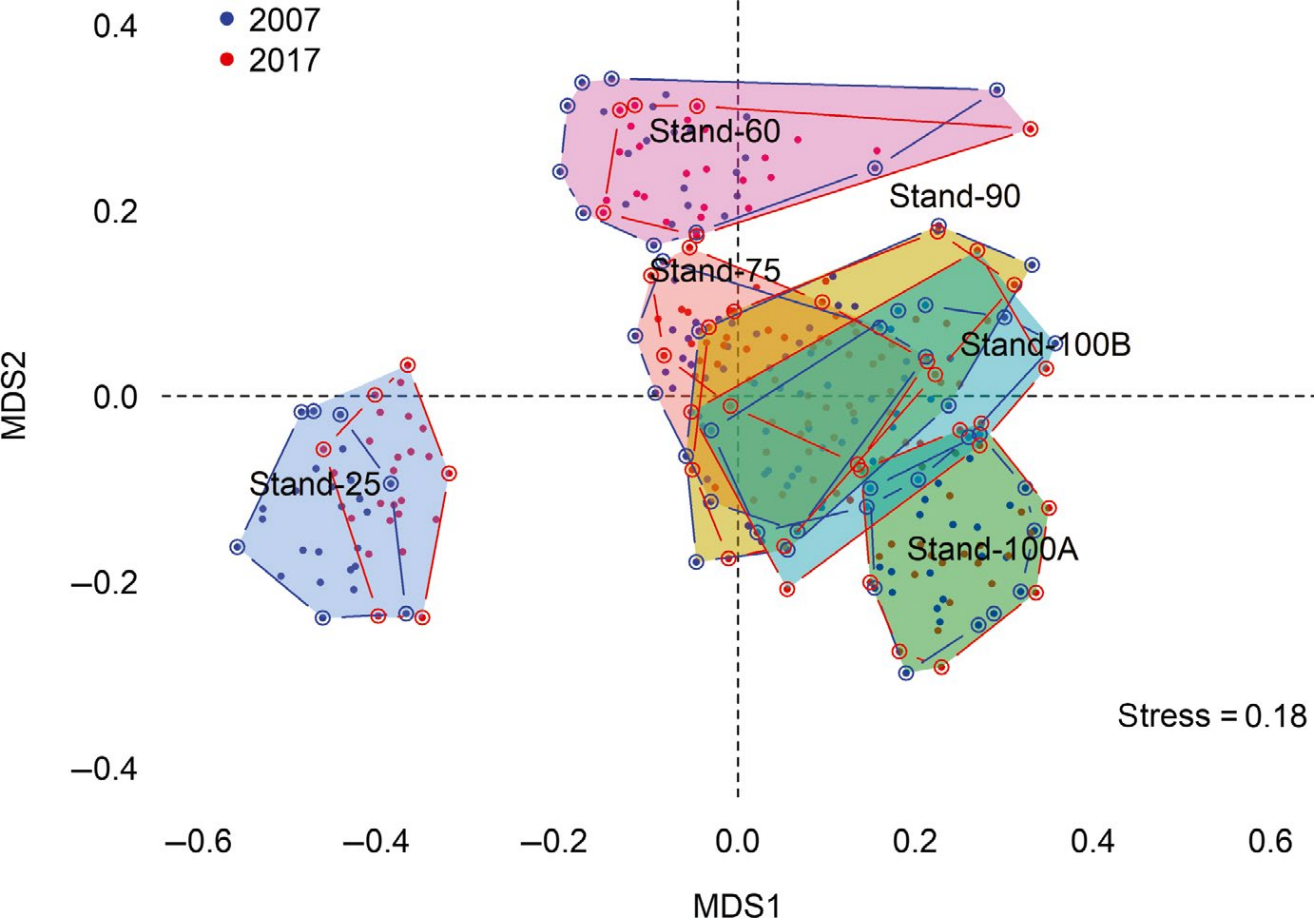
Nonmetric multidimensional scaling (nMDS) ordination of the species occurring in six stand ages (25, 60, 75, 90, 100A, and 100B) during two study years (2007 and 2017) in secondary forests in southern Brazil

**Figure 4:**
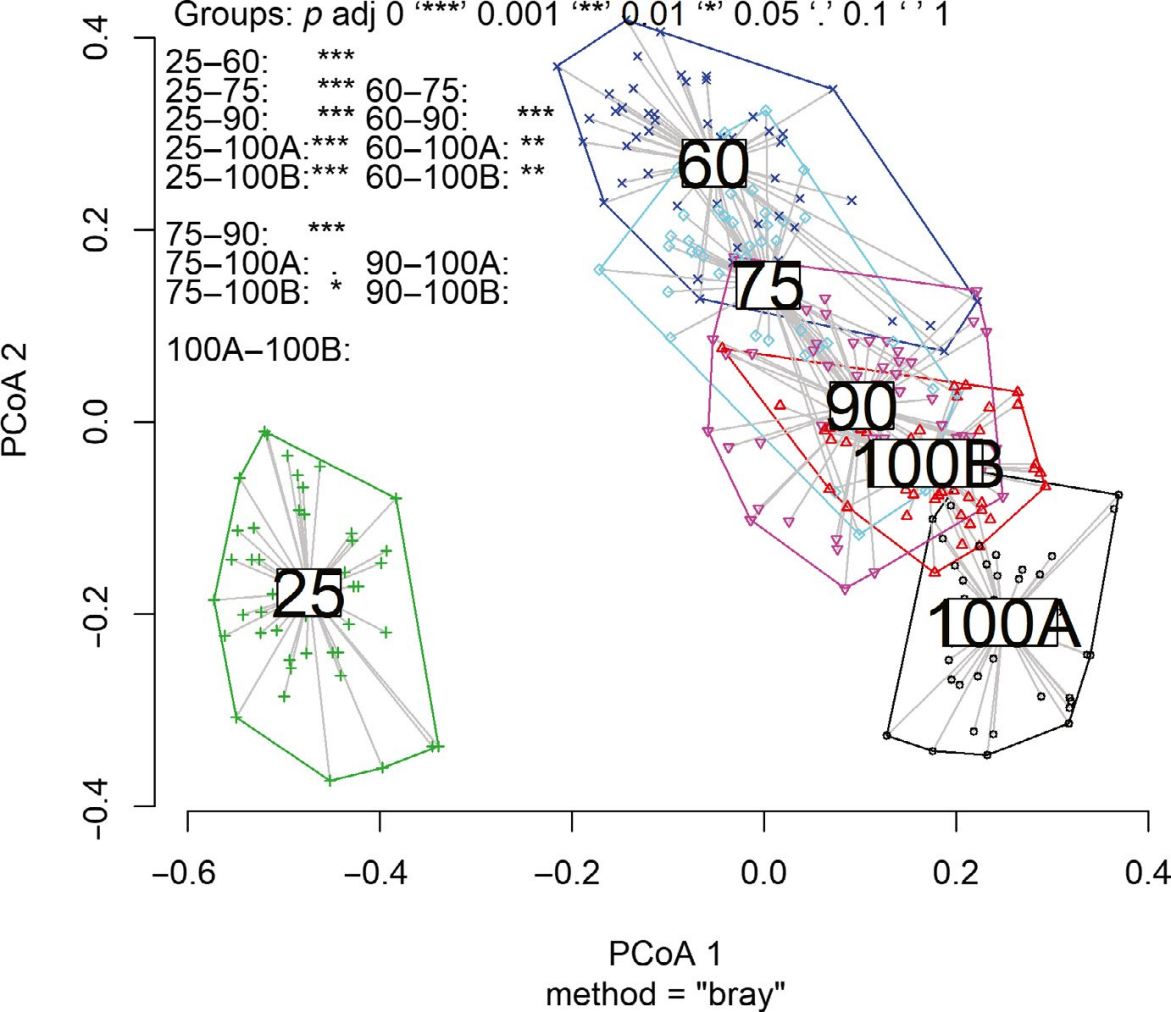
Multivariate homogeneity of groups dispersions (variances) and comparison between groups with Tukey's “Honest Significant Difference” method

The nMDS ordination with only two dimensions identified three nonoverlapping groups (Figure 3), with the younger stand (Stand‐25) being the most dissimilar to the others and more similar within itself. The stress values found for 2, 3, and 4 dimensions were 0.1780, 0.1359, and 0.1126, respectively, indicating that these ordinations are adequate for interpretation of multiple dimensions. Although the multivariate homogeneity test of group dispersions (Figure 4) indicated differences (*p* = .01), we believe that the balanced experimental design (even number of samples) makes PERMANOVA robust in relation to variance heterogeneity (Anderson, 2017). Stand‐25 had less variation among its subsamples but had a greater difference between study years and between older stands. The composition patterns showed a successional gradient where the younger stand was more distinguishable from the other stands (isolated to the left in the nMDS plot), and the other stands (Stand‐60, Stand‐75, Stand‐90, Stand‐100A, and Stand‐100B) were placed to the right side of the figure; following a chronological pattern of variation along the horizontal and vertical axes of the nMDS plot (Figure 3). That is, the nMDS suggests that stands appear to split chronologically to some extent, but not across a straightforward linear axis, reflecting stochastic changes in terms of compositional and multiple basins of attraction, providing evidence for a nonequilibrium community structure (Vandermeer et al., 2004).

### 3.4 β diversity between stand ages and study years

**Figure 5:**
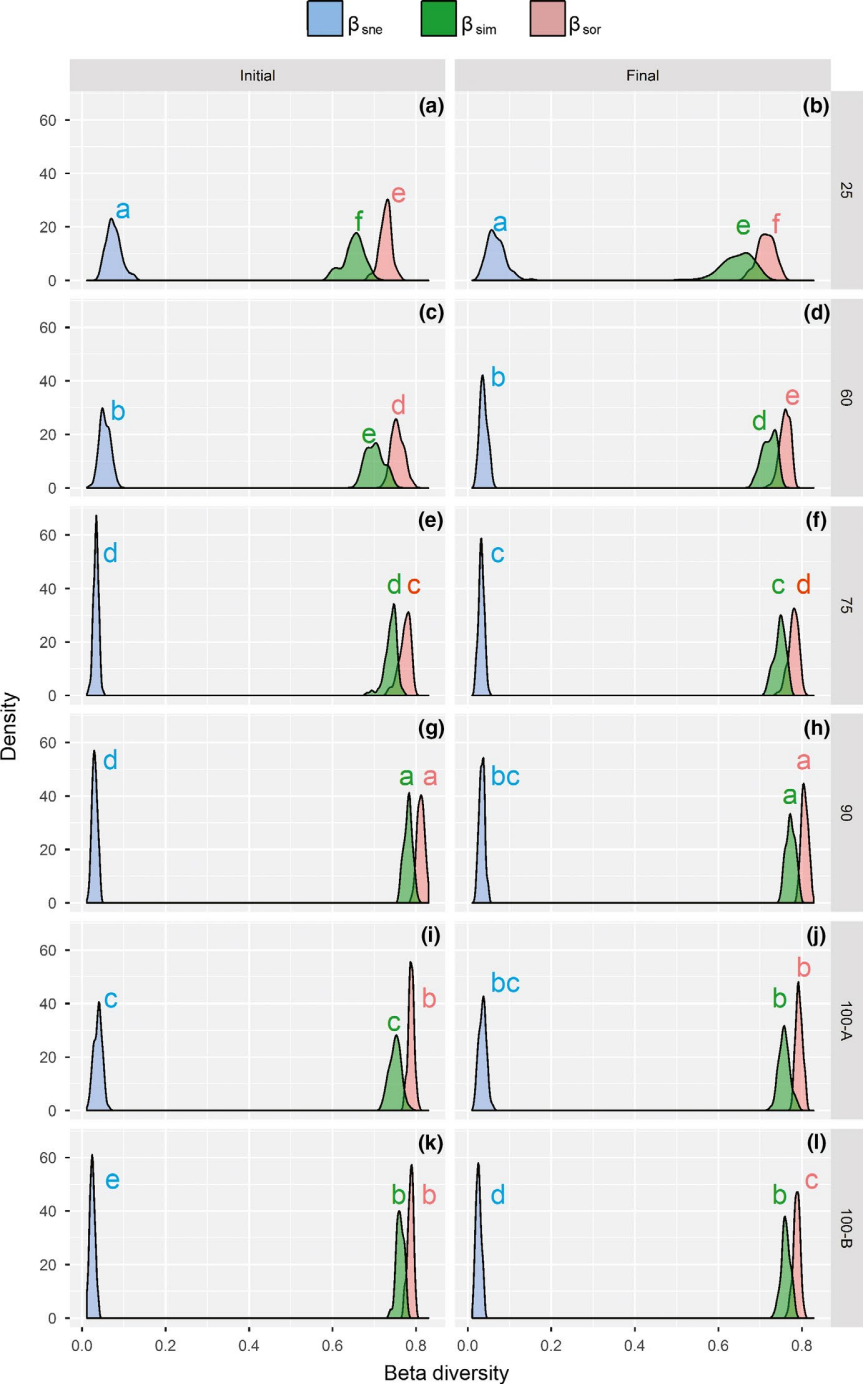
(a to l) Multiple‐site dissimilarities accounting for the components of beta spatial diversity, turnover (β_sim_), nestedness (β_sne_), and the sum of both values (β_sor_) in six stand ages (25, 60, 75, 90, 100A, and 100B), and two study years (2007 and 2017) in secondary forests in southern Brazil. Values with the same letter (for different stands) did not differ significantly by the Fisher's test using Bonferroni correction with *p* < .05

The partitioning of the Sørensen dissimilarity index (β_sor_) revealed a greater contribution of the turnover (β_sim_) component, compared to the nestedness (β_sne_) component, and to the overall dissimilarity for all stands and both study years (Figure 5). Proportionally, Stand‐25 and Stand‐60 (Figure 5a,c) presented higher nestedness than Stand‐75, Stand‐90, Stand‐100A, and Stand‐100B in the initial (2007) range (Figure 5e,g,i,k); this tendency was not consistent during the 10‐year interval (2017) when Stand 60 (Figure 5d) approached older stands (Figure 5h,j).

**Figure 6:**
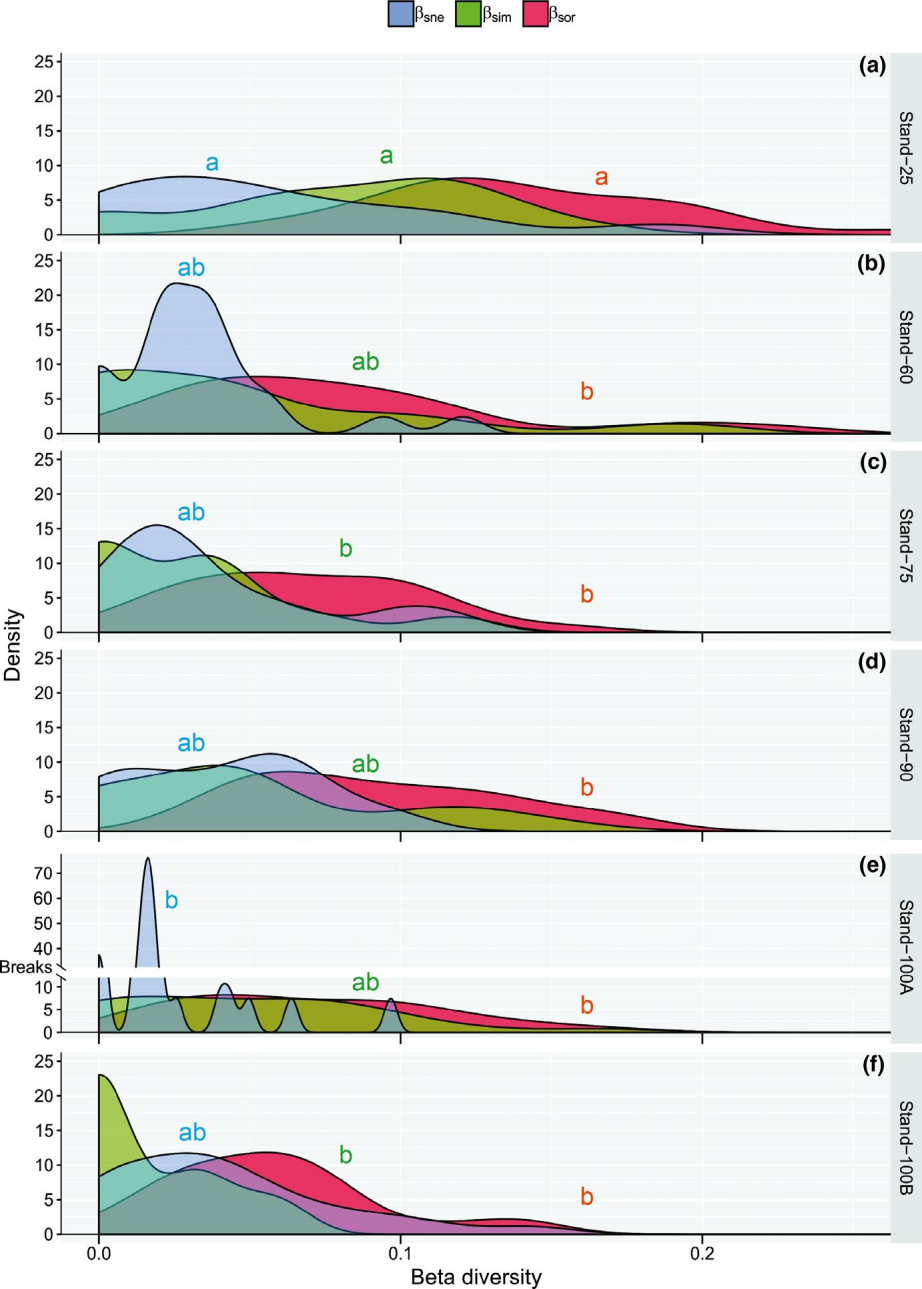
(a to f) Temporal changes over ten years in community composition according to the components of beta diversity, turnover (β_sim_), nestedness (β_sne_), and the sum of both values (β_sor_), in six stand ages (25, 60, 75, 90, 100A, and 100B) in secondary forests in southern Brazil. A scale break is on the *y*‐axis of the Stand‐100A to improve representation of data. Values with the same letter (for different stands) did not differ significantly by the Fisher's test using Bonferroni correction with *p* < .05

The comparison of temporal Sørensen dissimilarity index (β_sor_) between 2007 and 2017 showed that β_sor_ was higher and varied more among plots in Stand‐25 and did not differ in the older stands (Figure 6). The components of temporal β diversity, especially turnover (β_sim_), had the highest value in Stand‐25 (Figure 6a) compared to the other stands (Figure 6b–f). Moreover, the variation in the Sørensen dissimilarity index between stands (0.66–0.83; Figure 5) and between years (0–0.27; Figure 6) in absolute terms was similar, while in relative terms (variance/mean), temporal beta diversity showed much more variance than beta spatial diversity.

## 4 DISCUSSION

As hypothesized, we observed a high spatial‐temporal heterogeneity of the studied tree assemblages regarding species richness, structure, composition, diversity, and dynamics. In general, the youngest site was more floristically distinct, showed fewer species, a less structured stand, a lower spatial beta diversity but higher temporal beta diversity, and faster demographic rates than the oldest stands. Significant relationships were observed between the different parameters evaluated and the demographic rates (Figure S2), which are often additive effects. Taken together, this pattern suggests that forest succession is a complex process with a certain predictability in terms of structure and composition, with time postdisturbance being a fundamental predictor.

Despite the changes in the structure (abundance and basal area) and in the nestedness component of beta diversity, species richness, alpha diversity (Shannon), turnover component, and total beta diversity remained stable in the period of assessment (Table 2). However, that does not mean that there were no changes. The data collected on only two occasions limited the analysis of temporal interactions. As the demographic structure changes more abruptly after 25 years (Figure 2) and the species richness, diversity (Figure 1), composition (Figure 3), and beta spatial diversity (Figure 5) are more stable after 75 years, the demographics change and stabilize more rapidly than compositional diversity. In fact, evidence of a rapid recovery of species composition, ecological groups, biotic interactions, and functional characteristics in secondary forests is questionable (Chazdon, Careaga, Webb, & Vargas, 2003; Guariguata & Ostertag, 2001) since these types of forests house substantially less biodiversity compared to old‐growth sites (Turner, Wong, & Chew, 1997). Species composition may remain different in secondary forests for centuries (Finegan, 1996) or may never equate to that of a primary forest (Chazdon, 2008). For example, Liebsch et al. (2008) estimated that the Atlantic Forest needs up to three centuries to reach the proportion of species found in mature forests and significantly more time (between one and four thousand years) to reach the original levels of endemism. The progressive change in species composition (Sheil, 1999) indicates that a significant proportion of the successional trajectory may be explained by deterministic models of succession (Cequinel et al., 2018). On the contrary, differences between stands with the same postdisturbance recovering time (Stand‐100A and Stand‐100B) corroborates the evidence that secondary succession may not lead to convergence in species composition (Chazdon, 2008). This variation may be associated with different perturbations and related environmental variations such as soil conditions (Martins, Marques, Santos, & Marques, 2015), hydrological variation guided by topography (Zuleta et al., 2018), history of landscape use (Gross et al., 2018), differences in initial site conditions (Vandermeer et al., 2004), or stochastic processes that possibly operate along with environmental factors.

Considering that the size of the polygons in the hypothetical space of the nMDS (Figure 3) is proportional to the stochasticity, the intermediate supports (Stand‐60, Stand‐75, Stand‐90, and Stand‐100B) were more stochastic in the spatial rotation of species, while the extremes, Stand‐25 and Stand‐100A, were more consistent. We infer that a greater proportion of deterministic processes acted in their respective assemblies, and that the opposite positions had the greatest temporal dissimilarity (greater distance between polygons) suggesting that it is not the same environmental filter that acts in the assembly of their respective sets. The younger area showed a different composition. For the stages of 60 years or more, the composition variance was synchronized with PCoA2 (Figure 4) showing systematic changes. Systematic differences in temporal and spatial patterns suggest that there are changes in the relative strength of deterministic versus stochastic processes along gradients, results that cover the community assembly theory, identifying common mechanisms linking spatial and temporal patterns (Van Allen et al., 2017).

The demographic rates of changes in stands (Figure 2) were larger and more stochastic in the younger groups. The observed mortality (1.5%–2.7% per year) and recruitment (0.7%–2.6% per year) rates were similar to those seen in other studies conducted in tropical forests that ranged from 0.7% to 2.9% per year for mortality and from 0.4% to 5.1% per year for recruitment (Marques et al., 2009; Phillips et al., 1994; Rolim, Jesus, Nascimento, Couto, & Chambers, 2005). The dynamics rates were particularly higher in the youngest stand (Stand‐25), having a balance between mortality (2.7% per year) and recruitment (2.6% per year). Older stands (over 60 years) showed a more imbalanced dynamic in favor of mortality and basal area gain (Table 2; Figure 1). These patterns are often reported in the Atlantic Forest, suggesting a self‐thinning process (mainly of small trees) caused by an intense interspecific and intraspecific competition among trees (Valim et al., 2018; Oliveira‐Filho et al., 2007; Werneck & Franceschinelli, 2004).

The total spatial dissimilarity of the assemblages was driven mainly by species substitution, with both of these generally increasing with time (stand age) (Figure 5). Our results agree with those of the analysis of the components of beta diversity for wide geographic areas that showed turnover five times greater than nestedness (Soininen, Heino, & Wang, 2018). A high proportion of the turnover component may indicate a natural process of species substitution (Baselga & Orme, 2012). A higher proportion of nestedness in more recently disturbed areas shows a greater of the same species co‐occurring (Wright & Reeves, 1992). In this case, differences in species are probably related to multiple factors, such as the frequency of dispersion events, environmental heterogeneity, and biotic interactions (Soininen et al., 2018). The temporal dissimilarity (changes in species composition between the two sampling periods) was higher for the youngest stand, with the higher turnover rate driving species substitution (Figure 6). Trade‐off between spatial and temporal beta diversity occurs as stands grow older, so young stands show more temporal turnover of species than old stands, and, conversely, old stands show larger spatial turnover of species than young stands (Figure 6). Colonization generally dominates the initial successional change, and turnover rates decrease steadily at rates of species gain due to increasing competitive pressure and a decreasing pool of potential new colonists (Sheil et al., 2000). These results confirm that, as a consequence of demographic events in the studied secondary tropical forest, local extinction was surpassed by the arrival of new immigrant species. This indicates that disturbances have resulted in significant changes in species composition that are becoming more heterogeneous by gradually regaining diversity over time. Despite the relatively short period for a community of trees (10 years), the changes in the ecosystem reflected a wide variation in beta diversity (Figure 6). It is important to note the magnitude of the temporal fluctuations is likely to depend on the sampling interval and tree communities turn into decades (Van Allen et al., 2017). Thus, the patterns that we identify should be better exploited for a longer period of time, since we recognize that in this aspect the differences in temporal beta diversity for the period of a decade are not consistent with establish relations with the successional trajectory.

Our results demonstrate different aspects of secondary succession in a tropical hyper‐diverse forest inserted within a forest matrix that is relatively well preserved. The demographic rates and diversity recorded in the 10‐year interval indicate that the “rapid and dynamic process of species replacement and structural changes” takes place in younger stands (over time and space) and only over space in older stands. Such results reiterate the complexity and variability in forest succession in tropical ecosystems and can be used as benchmark for evaluating and monitoring the effectiveness of restoration measures to promote biodiversity and forest structural recovery in impacted tropical ecosystems that sustain the ecological processes critical for the maintenance of biodiversity.

## CONFLICT OF INTEREST

We declare we have no conflict of interests.

## AUTHORS' CONTRIBUTIONS

R. M. conceived the ideas and designed methodology; V. P. Z. and C. A. D. collected the data; C. A. D. and M. C. M. M. analyzed the data; M. C. M. M. corrected the manuscript, presented suggestions, and contributed in a relevant way in the introduction. P. H. corrected the manuscript, presented suggestions, and contributed in a relevant way in the discussions; R. M., V. P. Z., M. C. M. M., and P. H. reviewed critically the article. C. A. D. led the writing of the manuscript. All authors contributed critically to the drafts and gave final approval for publication.

## Data Availability

Data collection in 2007 and the second sampling in 2017 are available from the (https://ppbiodata.inpa.gov.br/metacatui/#view/PPBioAmOc.553.2).
