# Supplementary figures and images for: Spatial and temporal structure of diversity and demographic dynamics along a successional gradient of tropical forests in southern Brazil
Source: Ecol Evol. 2020 Mar 13;10(7):3164–77. doi: 10.1002/ece3.5816 (PMC7141045; doi:10.1002/ece3.5816)

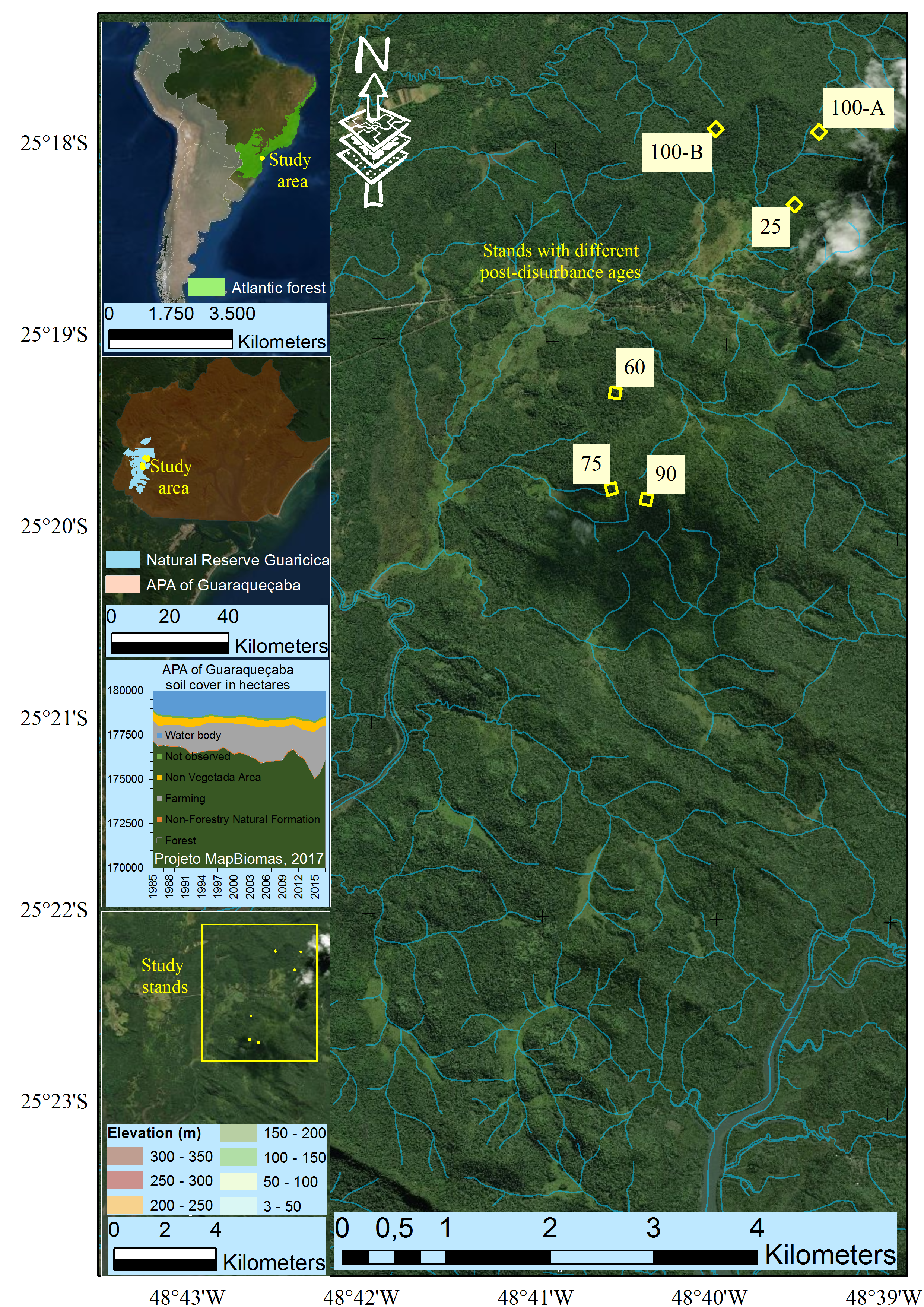

Supplement: Supplementary file 1 [file ECE3-10-3164-s001.tif]
