# Supplementary material for: Spatial and temporal structure of diversity and demographic dynamics along a successional gradient of tropical forests in southern Brazil
Source: Ecol Evol. 2020 Mar 13;10(7):3164–77. doi: 10.1002/ece3.5816 (PMC7141045; doi:10.1002/ece3.5816)
Supplement: Supplementary file 3 [file ECE3-10-3164-s003.docx]

Supplementary Table S1. Formulas described in common algebraic notation to calculate demographic rates in Atlantic forest remnants in southern Brazil.

| **Eq.** | **Demographic rates (% year^-1^)** | **Equation** | **Reference** |
| --- | --- | --- | --- |
| Eq. (1) | Mortality | $MORT RATE=\left[ 1-\left( 1-\frac{N_{0}-N_{1}}{N_{0}} \right)^{\frac{1}{t}} \right]\times100$ | Sheil et al. (1995) |
| Eq. (2) | Recruitment | $RECR RATE=\left[ 1-\left( 1-\frac{N_{r}}{N_{1}} \right)^{\frac{1}{t}} \right]\times100$ | Sheil et al. (2000) |
| Eq. (3) | Turnover | $TURN=\frac{\left( MORT RATE+RECR RATE \right)}{2}$ | Phillips and Gentry (1994) |
| Eq. (4) | Basal area loss | $BA.LOSS. RATE=\left\{ 1-\left[ \frac{\left( {BA}_{0}-{BA}_{m}+{BA}_{d} \right)}{{BA}_{0}} \right]^{\frac{1}{t}} \right\}\times100$ | Oliveira-Filho et al. (2007) |
| Eq. (5) | Basal area gain | $BA.GAIN.RATE=\left\{ 1-\left[ 1-\frac{({BA}_{r}+{BA}_{g})}{{BA}_{1}} \right]^{\frac{1}{t}} \right\}\times100$ | Oliveira-Filho et al. (2007) |
| Eq. (6) | Basal area turnover | $BA.TURN=\frac{\left( BA.LOSS. RATE+BA.GAIN.RATE \right)}{2}$ | Oliveira-Filho et al. (2007) |
| Eq. (7) | β_Sørensen_ - beta diversity | $\beta_{\mathrm{sor}}=\frac{b+c}{2a+b+c}$ | Sørensen (1948) |
| Eq. (8) | β_Simpson_ - turnover | $\beta_{\mathrm{sim}}=\frac{min(b,c)}{a+min(b,c)}$ | Koleff, et al. (2003) |
| Eq. (9) | β_sne_ - nestedness | $\beta_{\mathrm{sne}}=\beta_{\mathrm{sor}}-\beta_{\mathrm{sim}}=\frac{b+c}{2a+b+c} -\frac{\min\left( b,c \right)}{a+\min\left( b,c \right)}=\frac{\max\left( b,c \right)-\min\left( b,c \right)}{2a+\min\left( b,c \right)+\max\left( b,c \right)}\times\frac{a}{a+\min\left( b,c \right)}$ | Baselga (2010) |

*N_0_* = number of trees at the beginning of the measurement interval (*t*); *N_1_* = number of trees at the end of the measurement interval (*t*); *N_r_* = number of recruits; *BA_m_* = basal area by dead trees; *BA_d_* _=_ basal area decrease in that of survivors; *BA_r_* = basal area gained from recruits; BA_g_ = basal area increase in that of survivors; BA_0_ = basal area at the beginning of the measurement interval (*t*); BA_1_ = basal area at the end of the measurement interval (*t*); *a* = number of species in common between both sites; *b* = number of species occurring exclusively in the first location; *c* = number of species occurring exclusively in the second location.
