# Supplementary material for: Spatial and temporal structure of diversity and demographic dynamics along a successional gradient of tropical forests in southern Brazil
Source: Ecol Evol. 2020 Mar 13;10(7):3164–77. doi: 10.1002/ece3.5816 (PMC7141045; doi:10.1002/ece3.5816)
Supplement: Supplementary file 4 [file ECE3-10-3164-s004.docx]

**Supplementary Figure S1.** Location of the sampled stands with different post-disturbance ages (25, 60, 75, 90, 100A, and 100B) in Atlantic forest remnants in southern Brazil. For obtaining soil cover data we used Projeto MapBiomas (2017).

References:

Projeto MapBiomas (2017). *Coleção 3 da Série Anual de Mapas de Cobertura e Uso de Solo do Brasil*. Retrieved from [http://mapbiomas.org/map#](http://mapbiomas.org/map)
